# Efficient CRISPR/Cas9-Mediated Genome Editing Using a Chimeric Single-Guide RNA Molecule

**DOI:** 10.3389/fpls.2017.01441

**Published:** 2017-08-24

**Authors:** Haroon Butt, Ayman Eid, Zahir Ali, Mohamed A. M. Atia, Morad M. Mokhtar, Norhan Hassan, Ciaran M. Lee, Gang Bao, Magdy M. Mahfouz

**Affiliations:** ^1^Laboratory for Genome Engineering, Division of Biological Sciences, King Abdullah University of Science and Technology Thuwal, Saudi Arabia; ^2^Molecular Genetics and Genome Mapping Laboratory, Agricultural Genetic Engineering Research Institute, Agricultural Research Center Giza, Egypt; ^3^Department of Bioengineering, Rice University, Houston TX, United States

**Keywords:** genome engineering, CRISPR/Cas9, HDR, RNA-templated repair, gene editing

## Abstract

The CRISPR/Cas9 system has been applied in diverse eukaryotic organisms for targeted mutagenesis. However, targeted gene editing is inefficient and requires the simultaneous delivery of a DNA template for homology-directed repair (HDR). Here, we used CRISPR/Cas9 to generate targeted double-strand breaks and to deliver an RNA repair template for HDR in rice (*Oryza sativa*). We used chimeric single-guide RNA (cgRNA) molecules carrying both sequences for target site specificity (to generate the double-strand breaks) and repair template sequences (to direct HDR), flanked by regions of homology to the target. Gene editing was more efficient in rice protoplasts using repair templates complementary to the non-target DNA strand, rather than the target strand. We applied this cgRNA repair method to generate herbicide resistance in rice, which showed that this cgRNA repair method can be used for targeted gene editing in plants. Our findings will facilitate applications in functional genomics and targeted improvement of crop traits.

## Introduction

Recently, our ability to knock out genes in diverse eukaryotic species has dramatically increased due to advances in the development of site-specific nucleases (Kim and Kim, 2014; Voytas and Gao, 2014; Osakabe and Osakabe, 2015). The clustered regularly interspaced palindromic repeats (CRISPR)/CRISPR associated 9 (Cas9) system has been key in these advances, which are transforming biological and biomedical research. The CRISPR/Cas9 system is composed of the Cas9 endonuclease of *Streptococcus pyogenes* and a synthetic single-guide RNA (sgRNA), which directs the Cas9 protein to the user-selected genomic DNA sequence preceding the protospacer-associated motif (PAM) (Jinek et al., 2012; Mali et al., 2013). Engineering of a 20-nucleotide sequence in single or multiple sgRNAs, for single or multiple targets, respectively, has facilitated efficient genome engineering in transformable eukaryotic species (Gasiunas et al., 2012; Cong et al., 2013; DiCarlo et al., 2013; Li et al., 2013; Mali et al., 2013; Nekrasov et al., 2013; Aouida et al., 2015). The CRISPR/Cas9 system has been applied in diverse plant species for targeted mutagenesis (Li et al., 2013; Nekrasov et al., 2013; Sugano et al., 2014; Ali et al., 2015) and virus resistance (Zaidi et al., 2016). The CRISPR/Cas9 system generates site-specific double strand breaks (DSBs) at user-defined genomic sequences, which are repaired mainly through the error-prone non-homologous end joining (NHEJ) process or the more precise homology-directed repair (HDR) process (Puchta et al., 1996; Symington and Gautier, 2011; Onozawa et al., 2014; Sander and Joung, 2014; Voytas and Gao, 2014).

During natural homologous recombination (HR) that takes place in germ line cells during meiosis, homologous chromosomes exchange DNA segments resulting in genetic variation in a species. For engineering genomes, gene targeting commonly uses a type of HR to specifically alter the sequences of target genes, replace gene sequences, or add genes or parts of genes (Capecchi, 1989; Terada et al., 2002). In this method, genetic recombination takes place between the target chromosomal segment and introduced DNA, leading to a replacement of chromosomal segments or genes by the inserted DNA (Gloor et al., 1991; Donoho et al., 1998; Bibikova et al., 2003; Fauser et al., 2012). Recently, geminivirus replicons have been used to express the CRISPR/Cas9 machinery for the generation of targeted DNA DSBs and enhanced gene targeting (Baltes et al., 2014).

The process of NHEJ is predominant in plants, while HR is quite inefficient (Puchta et al., 1996; Symington and Gautier, 2011; Doudna and Charpentier, 2014; Steinert et al., 2016). Therefore, it is feasible to achieve targeted, small insertions or deletions (InDels) at user-specified loci, but more difficult to produce precise, user-defined alterations of the plant genomic sequence. Therefore, the development of efficient HR platforms will open a myriad of opportunities in targeted improvement of crop traits.

Multiple mechanisms and approaches have been used to manipulate NHEJ and HDR to gain control over the outcome of the repair process. Recent studies have shown that DNA repair mechanisms can use RNA as a template (Angeleska et al., 2007; Storici et al., 2007; Keskin et al., 2014, 2016; Zalatan et al., 2015; Meers et al., 2016). RNA templates have been shown to control DNA rearrangement in ciliates (Keskin et al., 2014), and synthetically delivered RNA was capable of mediating targeted DNA deletions. Thus, RNA-mediated genome rearrangement can utilize the DSB formation, and the DSBs are used to rearrange the DNA segments. Moreover, RNA-templated DNA synthesis has been in the repair of DSBs in *Saccharomyces cerevisiae* (Keskin et al., 2014) and in human cells (Zalatan et al., 2015). Notably, RNA synthesis can occur through other mechanisms besides reverse transcription. Given the ease of delivery of sgRNA molecules compared to DNA, here we tested whether a chimeric sgRNA (cgRNA) could serve dual functions as sgRNA for the generation of targeted DSBs and as a template for DNA repair via HDR.

In this study, we investigated the potential for using cgRNA repair templates to mediate DNA repair following the DSBs generated by the Cas9 endonuclease. We generated different DNA molecules that can be transcribed by the RNA Pol III promoter to produce different sgRNAs that can direct Cas9-mediated targeted DSBs and serve as a repair template for HDR. We applied this repair platform in rice protoplasts for targeted gene editing of *OsALS* (*Acetolactate synthase*) and for targeted insertion of the HA tag in *OsHDT701* (*Histone Deacetylase701*). We used cgRNAs that carried sgRNA to produce DSBs at the *OsALS* target site and also served as a repair template containing two simultaneous point mutations flanked by homology sequences. Similarly, we also targeted *OsHDT701* using an sgRNA to produce DSBs and to serve as a repair template that contained a 3XHA tag flanked by homology sequences. Amplicon deep sequencing showed the integration of the 3XHA tag in the rice *HDT701* locus and two substitutions in the rice *ALS* gene generated using CRISPR/Cas9-mediated homologous recombination in rice protoplasts. The repair template complementary to non-target strands showed higher DNA repair efficiency. We then used the cgRNA/Cas9 gene-editing platform to produce herbicide-resistant rice via *Agrobacterium*-mediated transformation and subsequent regeneration on bispyribac sodium containing medium. The quick and efficient production of herbicide resistant rice in this study demonstrates the applicability of this cgRNA/Cas9 platform for gene editing in crop species.

## Results

### Design and Construction of Chimeric gRNA Repair-Templates for Genome Editing

**FIGURE 1:**
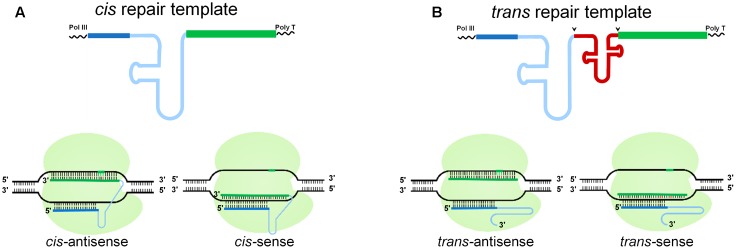
Schematic of RNA-templated DNA Repair. Pol III transcribed RNA was used for the gRNA. **(A)** In *cis* repair, the gRNA (blue) is attached directly to the repair template (green). **(B)** In *trans* repair, a tRNA (red) separates the gRNA and the repair template, which are processed into two separate RNA molecules; arrows indicate the cleavage positions. Below is a schematic of *cis* and *trans* repair at a target locus, where a target-specific gRNA produces a CRISPR/Cas9-mediated DSB that is repaired by HDR. The repair template is in antisense or sense orientation relative to the gRNA sequence. Dark blue line, 20-nt target specific gRNA; light blue line, scaffold RNA; green line, repair template; the small vertical line represents the nucleotide sequence, the green vertical lines represent the PAM sequence, the red vertical lines represent mutations introduced through template-mediated repair.

To investigate whether a cgRNA can be used as a template for DNA repair in plant cells, we designed several modalities with different architectures in sense and antisense orientations (**Figure 1**). The RNA templates were generated as a *cis* repair template, where the sgRNA and the repair template work as a single bifunctional molecule (**Figure 1A**), or as a *trans* repair template, where the sgRNA and the repair template were separated by a tRNA, which was further processed to give two RNA molecules (i.e., the sgRNA and the repair template) (**Figure 1B**) (Xie et al., 2015). The repair templates were designed in two orientations. The antisense orientation was complimentary to the non-target strand and the sense orientation was complementary to the target strand (**Figure 1**). Two different scaffold sgRNAs were tested during the *cis* repair, the scaffold RNA sequence used for rice (Xie et al., 2015) and the human scaffold RNA sequence as previously described (Chen et al., 2013; Zalatan et al., 2015). In our *cis*-repair designs, targeting of the RNA repair template to the DNA at the site of the DSBs or lesions was mediated by the Cas9 endonuclease. Therefore, Cas9 is serving a dual purpose; first as an endonuclease generating a targeted DSB, and second as a loading scaffold for an RNA template for RNA-mediated DNA repair. Therefore, we envisioned the use of a single, bifunctional cgRNA molecule for HDR (*cis* repair) and/or the availability of separate sgRNAs and repair molecules for DNA repair (*trans* repair). To prevent re-editing by the CRISPR/Cas9 machinery and to maximize the efficiency of RNA-templated DNA repair, we mutated the PAM site sequence by synonymous (silent) mutations in the repair template.

### cgRNA Molecules Mediate Repair of DSBs in Rice Protoplasts

**FIGURE 2:**
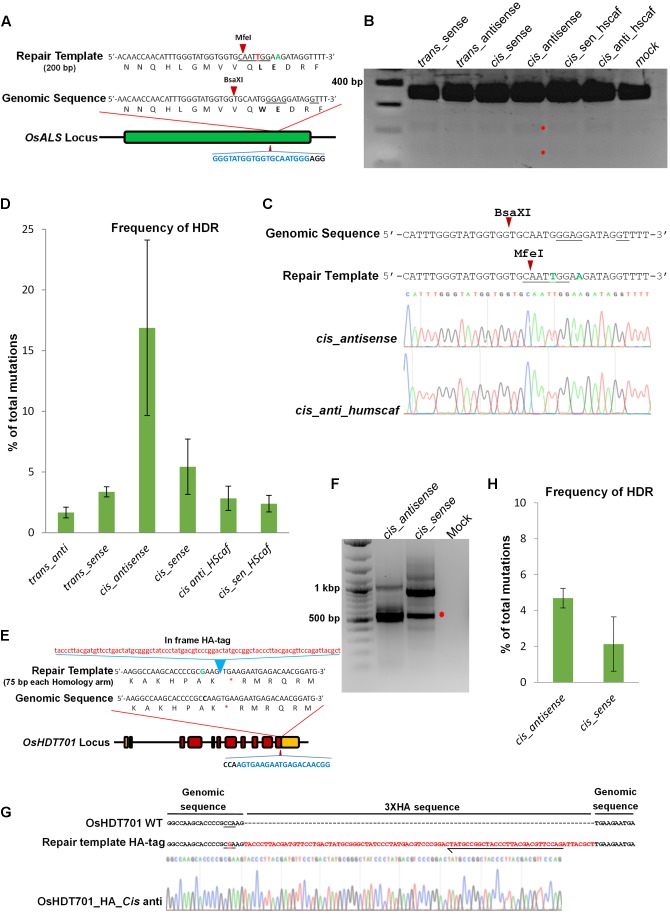
Homology-directed repair in rice protoplasts. **(A)** The target sequences in the *OsALS* (*LOC_Os02g30630*) locus. Blue nucleotides represent gRNA sequence and the PAM sequence is in black. The wild-type sequence has the *BsaXI* site underlined. Repair from the template introduces two substitutions, one in the PAM sequence G to A (green) and the other G to T (red). The second substitution destroys the *BsaXI* site and introduces an *MfeI* site (underlined). Arrows indicate an enzyme cutting site. **(B)** Genome-editing at the *ALS* locus in rice protoplasts. Rice protoplasts were transfected with the same gRNA and Cas9 but different repair templates, as indicated. DNA was extracted from protoplasts and enriched with *BsaXI* and PCR products were digested with MfeI to detect nucleotide substitutions. The red asterisks indicate the digested bands. **(C)** The selected PCR fragments were cloned and Sanger sequenced. For *cis-antisense*, 16 out of 96 (16.67% of total reads) have indels and 2 (12.5% of mutated reads) of these were repaired. For *cis-anti_humscaff*, 69 out of 253 (27.27% of total reads) have indels and 1 (3.70% of mutated reads) of these was repaired. **(D)** PCR was performed using genome-specific primers. Amplicons were deep sequenced and analyzed using CRISPResso software. The Ratio of HDR among total mutations was calculated. The *cis-antisense* template showed the highest repair efficiency. Error bars represent SE (*n* = 3). **(E)** The target sequences at the *OsHDT701* (*LOC_Os05g51830*) locus. Blue nucleotides represent the gRNA sequence and black nucleotides represent the PAM sequence. The repair template is 237 bp long including 87 bp of 3XHA-tag. **(F)** Protoplasts were transfected with HA-tagged repair templates as indicated. DNA was extracted and allele-specific PCR was conducted. Red asterisks indicate the expected PCR product size. **(G)** Gel elution was done and after cloning, Sanger sequencing was performed which showed the insertion of the HA-tag sequence in the genome. Black arrow represents the position of reverse primer used for allele-specific PCR. PAM sequence underlined. **(H)** PCR amplification was done using genome-specific primers from the transfected protoplast DNA. Amplicon deep sequencing was done and analyzed by CRISPResso software. *cis-*antisense showed higher efficiency compared to the *cis-sense* repair template. Error bars represent SE (*n* = 3).

The activities of different cgRNA-molecules were first tested in rice protoplasts to determine their capability to induce DSBs and simultaneously serve as a repair template to mediate HDR. We targeted the *OsALS* (*Acetolactate synthase*) locus (LOC_Os02g30630) for gene editing (**Figure 2A**). We transfected Nipponbare rice protoplasts with DNA molecules carrying *Ubi::Cas9* and *U3::cgRNAs* containing specificities for targeting and repair of the *ALS* gene. The protoplast DNA enriched for InDels by digesting with *BsaXI*. Next, we performed polymerase chain reaction/restriction enzyme digestion (PCR/RE; **Figure 2B**). All of the cgRNA molecules were fully functional in the generation of DSBs and the repair efficiency was highest in the *cis-*antisense repair templates when compared to the other architectures. Next, we performed Sanger sequencing to validate our PCR/RE data. Our Sanger sequencing data corroborated the PCR/RE data and showed that the cgRNA in the *cis-*antisense architecture was capable of mediating precise DNA repair (**Figure 2C**). To further validate these results, we performed PCR using genome-specific primers and amplicon deep sequencing. Our data were analyzed using CRISPResso software (Pinello et al., 2016), which revealed that the average of three independent experiments showed the highest repair percentage (16.88% HDR) for the repair templates with the *cis-*antisense architecture (**Figure 2D**).

Next, we tested the potential for this system to introduce larger DNA fragments by fusing the 3XHA tag at the *OsHDT701* locus in the rice genome. We used a cgRNA specific to *OsHDT701* to generate DSBs and to serve as a repair template containing the 3XHA-tagged sequence flanked by 75 bp of homology sequence for transfections in rice protoplasts (**Figure 2E**). We then used allele-specific PCR to test for insertion of the 3XHA tag. The allele-specific PCR data clearly showed the integration of the HA tag at the *OsHDT701* locus (**Figure 2F**), which was further validated by Sanger sequencing (**Figure 2G**). Next, we performed deep sequencing using genome-specific primers and found that the efficiency of HDR for the *cis-*antisense template was 4.69%, which was higher than that of the *cis-*sense architecture (2.13%) (**Figure 2H**). The introduction of the silent mutation in PAM in the repair template prevents re-editing of the target as evidenced by the recovery of HDR events lacking any Cas9-recutting activities. We observed that most of the mutations were deletions (Supplementary Figures 1A,B). Since we transfected DNA molecules into the protoplast cells, it is possible that DNA or DNA/RNA molecules were used as repair templates. We tested this system by using cgRNA as RNA molecules and transfected rice protoplasts with ribonucleoprotein complexes with the cgRNA as RNA only and the Cas9 protein (Supplementary Figures 2A,B). The ribonucleoprotein complex was capable of inducing DSBs and simultaneous DNA repair. Although the repair efficiency was less than that observed when DNA molecules were used for transfections, none the less, the cgRNA could be used for targeted DNA repair of DSBs (Supplementary Figures 2C,D). Furthermore, amplicon deep sequencing showed the highest HDR for the *cis-*antisense templates (Supplementary Figure 2D). In all of our experiments, and specifically with the ribonucleoprotein transfections, the mutation rate through NHEJ was much higher than through HDR (Supplementary Figure 2E). Therefore, further studies are needed to conclusively establish that RNA molecules can serve as templates for RNA-mediated DNA repair in plant cells.

### cgRNA/Cas9-Mediated Targeted Gene Editing to Confer Herbicide Resistance in Rice

To test the applicability of the cgRNA-mediated genome editing method, we selected the *ALS* gene for mutagenesis and repair to produce plants resistant to bispyribac sodium (BS) herbicide. The enzyme acetolactate synthase (ALS) catalyzes the initial step of the biosynthesis of the branched-chain amino acids leucine, isoleucine, and valine (Chipman et al., 1998). ALS is the primary target site of action for at least four structurally distinct classes of herbicides (sulfonylureas, imidazolinones, triazolopyrimidine sulfonamides, and pyrimidinyl carboxy herbicides) (Shimizu et al., 2005). BS belongs to the pyrimidinyl carboxy herbicide group. Rice BS tolerance is linked to two point mutations in the *ALS* gene: a tryptophan (TGG) to leucine (TTG) change at amino acid 548 (W548L), and a serine (AGT) to isoleucine (ATT) change at amino acid 627 (S627I) (Mazur et al., 1987; Endo et al., 2007). However, a single point mutation (W548L) is enough to confer resistance in rice (Endo et al., 2007).

**FIGURE 3:**
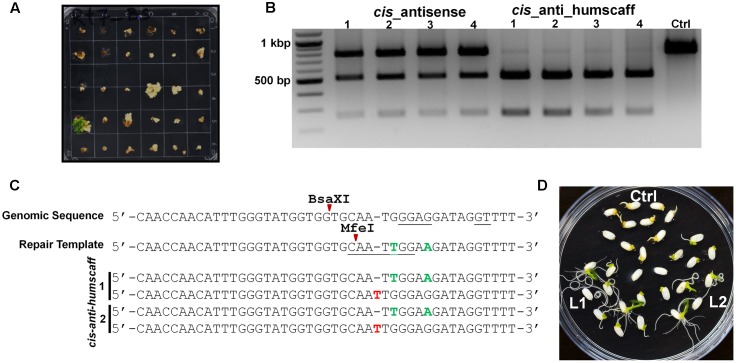
RNA-templated DNA Repair produced Herbicide-resistant Plants. **(A)** In *OsALS* Tryptophan (TGG) to leucine (TTG) at amino acid 548 (W548 L) showed high bispyribac sodium (BS) herbicide resistance. The transformed calli were regenerated with BS. The seedlings transformed with *cis_anti_humscaff* repair template have stronger resistance and are well-established. **(B)** DNA was extracted from young seedlings and mutations were detected by MfeI digestion. The plants with desired mutations showed digested bands and the control showed an MfeI-resistant undigested band. **(C)** These amplicons were cloned and Sanger sequenced. The seedlings with the *cis_anti_humscaff* repair template showed repair (green nucleotides). Red nucleotides show other Indels. **(D)** Seeds from *cis_antis_humanscaff* repair template BS-resistant plants were germinated on ^1^/_2_MS+0.75 μM BS and showed clear BS resistance. These resistance plants were Sanger sequenced and showed homozygous repair (Supplementary Figure 3).

We employed our cgRNA platform to generate BS resistance. We used *Agrobacterium*-mediated rice transformation and selected proliferating calli for regeneration on medium with 0.75 μM BS (**Figure 3A**). Only the mutated callus cells survived and regenerated as shoots. The plantlets were further analyzed by PCR/RE and Sanger sequencing (**Figures 3B,C**). For each cgRNA architecture, approximately 700 calli were transformed. After selection, approximately 15 plantlets were regenerated for the *cis_anti_humscaff* template (HDR was 2.14%). No regeneration was observed with the other architectures. For the *cis_antisense* template, approximately five plantlets were recovered, but these were false positives. To further confirm the herbicide resistance, we screened the seeds of progeny on 0.75 μM BS medium and confirmed that all progeny were resistant to BS (**Figure 3D**). We genotyped these plants and found that some were homozygous (Supplementary Figures 3A,B), indicating the applicability of this platform for targeted engineering of plant genomes.

## Discussion

Significant improvements in HDR are required to ensure widespread application of CRISPR/Cas9 genome engineering, specifically in genomic medicine and targeted improvement of crop traits (Doudna and Charpentier, 2014; Hsu et al., 2014; Verma and Greenberg, 2016). Approaches to increase the efficiency of HDR include titration of the concentrations of sgRNA and Cas9 endonuclease, using small molecules to inhibit NHEJ, and performing editing at a specific stage of the cell cycle (Bi et al., 2014; Lin et al., 2014; Chu et al., 2015; Wang et al., 2015).

Double strand breaks have been shown to induce the generation of small RNA molecules (diRNA) at or around the DSB site in plants and animals (Yamanaka and Siomi, 2014). Intriguingly, the small RNA pathways were implicated in this process, and the loss of function of key components of these epigenetic pathways abrogated such repair (Yamanaka and Siomi, 2014). However, it remains to be determined whether these diRNAs can serve as templates for DNA repair. RNA molecules have been shown to mediate DNA rearrangements in ciliates and to serve as templates for DNA synthesis in the repair of DSBs in *Saccharomyces cerevisiae* (Keskin et al., 2014). RNA oligos complementary to the broken ends were used in these studies. These studies indicated that genetic information can be transferred from RNA to DNA. One possibility is that the RNA molecules serve as a scaffold bringing together two DNA molecules and facilitating the repair. Exchange of genetic information between RNA and DNA and pairing between RNA and DNA has been shown *in vitro* and *in vivo* in RNA–DNA hybrids (Huertas and Aguilera, 2003).

In this study, we used the Cas9 endonuclease for the targeted generation of DSBs in the genome and for targeted delivery of RNA molecules homologous to the DNA ends around the DSBs to test whether these substrates could be used for precise DNA repair. We used cgRNAs containing sgRNA for targeted generation of DSBs and as RNA repair templates. These cgRNAs were designed with different architectures to test different fusions including *cis* and *trans* repair. Our data demonstrated that cgRNAs can serve as a guide for the Cas9-mediated generation of DSBs as well as an RNA template for subsequent DNA repair. Specifically, cgRNA in the c*is-*antisense architecture can be used to generate precise gene editing with reasonable efficiency in rice protoplasts.

We used our cgRNA/Cas9 system for the precise introduction of an HA tag flanked by a 75 bp homology to target the *OsHDT701* locus in the rice genome. Allele-specific PCR and amplicon deep sequencing confirmed the successful introduction of the HA tag. However, a more rigorous analysis of the effect of the length of the RNA template from the PAM proximal and PAM distal sites is needed to determine its effects on the efficiency of genome editing. It should be noted that we were not able to recover rice plants with the introduced HA tag. Therefore, further studies are needed to determine the possibilities, limitations, and potential improvements of this cgRNA/Cas9 system.

We also designed a repair RNA template of 200 bp in length with two substitutions to target *OsALS*. We used these substitutions to destroy the *BsaXI* site and to introduce the *MfeI* site for quick detection by PCR/RE analysis. Our results showed the highest DNA repair efficiency for the c*is-*antisense templates in rice protoplasts. Furthermore, to show the applicability of this cgRNA/Cas9 system for gene editing, we edited the *ALS* gene in the rice genome to confer tolerance to BS.

The precise molecular mechanisms of the cgRNA/Cas9-mediated gene-editing platform are not known and warrant further investigation. Because different substrates could be used for DNA repair, including ssDNA, dsDNA, RNA–DNA hybrids, and RNA only, many possible mechanisms could be envisioned for cgRNA/Cas9-mediated gene editing. As we delivered the templates in a DNA form for generation of cgRNA, we cannot exclude the possibility that some of the repair observed during protoplast transformation was due to DNA or DNA–RNA hybrids. It might be possible that the large amount of plasmid DNA available during protoplast transformation was used as a DNA repair template. However, different cgRNA architectures, with the same DNA molecules, produced drastically different editing efficiencies. For example, cgRNA with a complementary sequence to the target strand failed to mediate targeted gene editing. To exclude the possibility of plasmid DNA used for repair, we used *in vitro* transcribed RNA as a repair template. The ribonucleoprotein transfections in the protoplasts indicated that RNA alone can mediate targeted editing, although with reduced efficiency, showing that the cgRNA/Cas9 system was indeed capable of mediating precise repair.

In summary, we used cgRNA as a guide for the generation of DSBs and as an RNA template for subsequent DNA repair. We detected successful substitutions in the *OsALS* locus and insertion of an HA tag in rice protoplasts. Furthermore, we demonstrated the applicability of this cgRNA/Cas9 system for gene editing by editing the *ALS* gene in rice to produce herbicide-tolerant plants. Our platform depends on the CRISPR/Cas9 machinery to deliver the cgRNA repair template simultaneously as a single RNA molecule. Therefore, this platform could facilitate the generation of non-transgenic genome-edited crops using the RNP molecules. This cgRNA-templated DNA repair system may facilitate the generation and screening of protein variants for functional studies and for targeted improvement of crop traits.

## Materials and Methods

### Vector Construction and Template Design

*Oryza sativa* L. ssp. *japonica* cv. Nipponbare was used for all experiments. pRGE32 and pRGEB32 vectors were used for protoplast and callus transformations, respectively (Xie et al., 2015). The expression of Cas9 and the cgRNAs were driven by the *OsUbiquitin* and *OsU3* promoters, respectively. ALS catalyzes the initial step in the biosynthesis of the branched-chain amino acids leucine, isoleucine, and valine (Chipman et al., 1998). The *ALS* locus in *Oryza sativa* L. ssp. *japonica* cv. Nipponbare is (LOC_Os02g30630). A change from tryptophan (TGG) to leucine (TTG) at amino acid 548 (W548L) resulted in higher herbicide resistance than a change from serine (AGT) to isoleucine (ATT) at amino acid 627 (S627I). The cgRNA was designed to target the genomic sequence between 1,721 and 1,743 bp (5′-GGGTATGGTGGTGCAA**TGGGAG**G-3′); the underlined AGG represents the PAM sequence. To mutate this sequence, the 200 bp template contained a point mutation that converts tryptophan (TGG) to leucine (TTG) at amino acid 548 (W548L), shown in bold red font. This mutation generated a recognition site for the restriction enzyme *Mfe*I (CAATTG, shown in red font), which allowed rapid detection of the RNA-template repair event. To alter the PAM sequence, a silent mutation was inserted directly next to the leucine codon (shown in bold black font), which converts GAG to GAA, both of which translate to glutamate. This mutation destroyed the *BsaX*I site. For the *trans*-repair template, a **polycistronic tRNA-gRNA** (PTG; Xie et al., 2015) fragment was synthesized containing the *OsALS* target gRNA—Scaffold RNA—pre-tRNA—Homology sequence (with mutations)—Terminator. Two PTG fragments were synthesized, one with a sense homology sequence and the other with an antisense homology sequence respective to the gRNA sequence. To test the potential of the *cis*-repair template, the repair template was inserted next to the gRNA scaffold sequence. The fragments were also synthesized in a sense and antisense orientation. The human scaffold RNA was used in another experiment to determine if there were differences between the human and yeast scaffold RNA sequences.

To test the potential of using this system to insert larger DNA fragments, we attempted to fuse a 3XHA tag at the *OsHDT701* locus (LOC_Os05g51830) in the rice genome. We used a cgRNA repair template with a 3XHA tag flanked by a 75 bp homology sequence. The gRNA sequence is 5′-GCCAAG**TGA**AGAATGAGACAACGG-3′. The PAM site (underlined) was silently mutated from GCC to GCG, both of which translated for alanine. The red font represents the mutated nucleotide and the bold font represents a stop codon. The PTG fragment was synthesized as *cis*-repair in both the sense and antisense orientation.

### Protoplast Isolation and Transformation

Protoplast isolation and transfection was performed as previously described (Shan et al., 2014). Ribonucleoprotein transfections were performed as described by Woo et al. (2015). RNA was synthesized by T7 *in vitro* transcription using the Thermo Scientific TranscriptAid T7 high yield transcription kit (#K0441). Two simultaneous *DNase*1 treatments were done, each followed by purification using the MEGAclear transcription clean-up kit (AM1908). After ensuring the complete DNA degradation by PCR analysis, RNA was loaded on the gel to check the quality and integrity and subsequently used for protoplast transfection. After the transfections, protoplasts were incubated at room temperature for 24–48 h. The DNA was extracted using the CTAB method (Butt et al., 2014) and further analyzed to detect the edited events.

### Rice Transformation

*Agrobacterium*-mediated rice transformation was performed as described (Hiei and Komari, 2008). For the selection of herbicide resistance, rice calli were regenerated on media supplemented with 0.75 μM BS (32967 Sigma-Aldrich). After 2–3 weeks, transgenic seedlings were transferred to sterile plastic containers containing fresh rooting medium and grown for 2–3 weeks before being transferred into soil. Transgenic rice plants were grown in a greenhouse at 28°C.

### Amplicon Sequencing

Using gene-specific primers, approximately 300 bp PCR amplicons were generated and purified, and subsequently subjected to amplicon deep sequencing. The library was prepared using the Illumina TruSeq Nano DNA Library Prep kit according to the manufacturer’s instructions. Libraries were run on the MiSeq platform. The sequencing data were analyzed using CRISPResso software (Pinello et al., 2016).

## Author Contributions

MaM and GB conceived the study. MaM and HB designed the experiments. HB, AE, ZA, NH, and CL, performed the experiments. Data analysis was performed by MA and MoM. HB and MaM wrote the paper.

## Conflict of Interest Statement

The authors declare that the research was conducted in the absence of any commercial or financial relationships that could be construed as a potential conflict of interest.
