# Supplementary material for: Efficient CRISPR/Cas9-Mediated Genome Editing Using a Chimeric Single-Guide RNA Molecule
Source: Front Plant Sci. 2017 Aug 24;8:1441. doi: 10.3389/fpls.2017.01441 (PMC5573723; doi:10.3389/fpls.2017.01441)
Supplement: Supplementary file 1 [file Data_Sheet_1.DOCX]

**Supplementary material**

**Efficient CRISPR/Cas9-mediated genome editing using a chimeric single-guide RNA molecule**

Haroon Butt^1^, Ayman Eid^1^, Zahir Ali^1^, Mohamed Atia^2^, Morad Mokhtar^2^, Norhan Hassan^1^, Ciaran Lee^3^, Gang Bao^3^, and Magdy M. Mahfouz^1, *^

^1^Laboratory for Genome Engineering, Division of Biological Sciences, 4700 King Abdullah University of Science and Technology, Thuwal 23955-6900, Saudi Arabia, ^2^Agricultural Genetic Engineering Research Institute (AGERI), Giza, Egypt, ^3^Department of Bioengineering, Rice University, Houston, TX 77030, USA

***Correspondence:** Magdy M. Mahfouz (Magdy.mahfouz@kaust.edu.sa)

 Key words: Genome engineering, CRISPR/Cas9, HDR, RNA-templated repair, gene editing

**Suppl. Fig. 1. Homology-Directed Repair in Rice Protoplasts using Plasmid Transfections at *ALS* locus.** **(A)** Example figures from one of the replications for mutation position distribution of NHEJ by CRISPResso software. The values represent all NHEJ events percent of total number of reads. Colored arrows indicate insertions, deletions and substitutions. **(B)** Example figures from one of the replications for mutation position distribution of HDR by CRISPResso software. The percent value showed the all events of HDR by total number of reads.

**Suppl. Fig. 2. Homology-Directed Repair Using RNP Transfection in Rice Protoplasts at *ALS* locus.** RNAs were synthesized by T7 *In vitro* transcription. To ensure complete DNA degradation, two simultaneous DNase treatments were done, followed by RNA purifications. For *trans*-repair, the RNA fragment of gRNA and the homologous sequence separated by tRNA was used and for *cis*-repair RNA fragment of gRNA and homologous sequence was used for transfections as described in Fig. 1. For DNA-only repair, the PCR product of the homologous sequence was used for transfection. **(A)** After two DNase treatments, each followed by purification, PCR was done using RNA as the template. No PCR product was observed after DNase treatment. Plasmid DNA was used as the positive control **(B)** RNA (1 µg) was run on an agarose gel to check the integrity of the RNA. **(C)** Cas9 protein was premixed with gRNA, incubated and used for protoplast transfections. The DNA was extracted from protoplasts and allele-specific PCR was done. The 308-bp amplicon showed a two-nucleotide substitution in the genome. No substitution was observed in trans-sense repair template, as the gRNA probably did not work. Additionally these substitutions were observed in samples where only the gRNA and Cas9 were used. **(D)** Deep sequencing of amplicons was conducted. The HDR percentage was calculated dividing by the total number of mutations. The highest values for HDR were observed for DNA-only but among RNA-only repair templates, *Cis-*antisense showed the highest HDR. The Z-score analysis indicates significant difference between the samples gRNA-only and *Cis-*antisense. **(E)** Comparison of mutation efficiency of RNP with plasmid transfections in rice protoplasts. The NHEJ values are much higher in RNP transfections.

**Suppl. Fig. 3. RNA-templated DNA Repair produced Herbicide-resistant Plants. (A)**  Herbicide-resistant plants (T2 generation) grow in greenhouse. Seedlings of c*is-anti-humscaff* were selected on ^1^/_2_MS+0.75 µM BS and transferred to soil. **(B)** Plants were genotyped by Sanger sequencing and show a two-nucleotide substitution.
